# BKV Agnoprotein Interacts with α-Soluble *N*-Ethylmaleimide-Sensitive Fusion Attachment Protein, and Negatively Influences Transport of VSVG-EGFP

**DOI:** 10.1371/journal.pone.0024489

**Published:** 2011-09-12

**Authors:** Mona Johannessen, Mari Walquist, Nancy Gerits, Marte Dragset, Anne Spang, Ugo Moens

**Affiliations:** 1 Research Group of Host-Microbe Interactions, Department of Medical Biology, Faculty of Health Sciences, University of Tromsø, Tromsø, Norway; 2 Biozentrum, University of Basel, Basel, Switzerland; University of Minnesota, United States of America

## Abstract

**Background:**

The human polyomavirus BK (BKV) infects humans worldwide and establishes a persistent infection in the kidney. The BK virus genome encodes three regulatory proteins, large and small tumor-antigen and the agnoprotein, as well as the capsid proteins VP1 to VP3. Agnoprotein is conserved among BKV, JC virus (JCV) and SV40, and agnoprotein-deficient mutants reveal reduced viral propagation. Studies with JCV and SV40 indicate that their agnoproteins may be involved in transcription, replication and/or nuclear and cellular release of the virus. However, the exact function(s) of agnoprotein of BK virus remains elusive.

**Principal Findings:**

As a strategy of exploring the functions of BKV agnoprotein, we decided to look for cellular interaction partners for the viral protein. Several partners were identified by yeast two-hybrid assay, among them α-SNAP which is involved in disassembly of vesicles during secretion. BKV agnoprotein and α-SNAP were found to partially co-localize in cells, and a complex consisting of agnoprotein and α-SNAP could be co-immunoprecipitated from cells ectopically expressing the proteins as well as from BKV-transfected cells. The N-terminal part of the agnoprotein was sufficient for the interaction with α-SNAP. Finally, we could show that BKV agnoprotein negatively interferes with secretion of VSVG-EGFP reporter suggesting that agnoprotein may modulate exocytosis.

**Conclusions:**

We have identified the first cellular interaction partner for BKV agnoprotein. The most N-terminal part of BKV agnoprotein is involved in the interaction with α-SNAP. Presence of BKV agnoprotein negatively interferes with secretion of VSVG-EGFP reporter.

## Introduction

*Polyomaviridae* are non-enveloped viruses containing a circular double stranded DNA genome of approximately 5,000 base pairs. Polyomaviruses infect several species, including birds, rodents, bovine, monkeys and human [1]. So far, eleven polyomaviruses have been found in humans. The first two were discovered in 1971, and named BK virus and JC virus after the initials of the patients from which they were originally isolated [2]. Two monkey polyomaviruses, lymphotropic polyomavirus (LPV) and simian virus 40 (SV40) also circulate among humans as indicated by the presence of antibodies against these viruses [3]. Moreover, in human leukoencephalopathy cases, SV40 and LPV DNA were detected in tonsils and in peripheral blood, respectively [4], [5]. A few years ago, two novel polyomaviruses, designated KI virus and WU virus, were found in human nasopharyngeal aspirates [6], [7], while a third, not previously described polyomavirus in Merkel cell carcinoma was isolated and accordingly named Merkel cell polyomavirus [8]. Last year, another three new human polyomaviruses were reported. Two of them were isolated from skin swabs, and referred to as human polyomavirus-6 (HPyV6) and -7 (HPyV7) [9]. The third virus was identified in spicules of trichodysplasia spinulosa (TS) and was named trichodysplasia spinulosa-associated polyomavirus (TSV) [10]. Finally, this year another human polyomavirus was detected in a kidney transplant patient, and named HPyV9 [11]. Given the rapid discovery of more and more of these viruses, it becomes very important to understand how the individual viral proteins interfere with host cell functions.

The most studied polyomaviruses are JCV, BKV and SV40. The primary infection of the two former typically occurs in childhood. Both viruses then establish a latent infection in kidney and peripheral blood in 35–85% of the population worldwide [1]. Asymptomatic reactivation of BKV may occur in 5% of the healthy individuals and during pregnancy. Although apparently harmless in immunocompetent hosts, BKV and JCV can cause diseases in immunocompromised individuals. BKV is a significant pathogen in kidney transplant patients, causing polyomavirus-associated nephropathy, which may result in renal allograft loss, while in bone-marrow recipients, BKV can cause hemorrhagic cystitis [12], [13]. JCV is the etiological agent of progressive multifocal leukoencephalopathy [14].

Phylogenetic studies have shown that BKV, JCV and SV40 are more closely related compared to the other human polyomaviruses [8], [15]. The polyomavirus genome of SV40, JCV and BKV is divided into three functional parts consisting of an early and a late gene region separated by a non-coding control region (NCCR). The NCCR contains the origin of replication as well as the promoter-enhancer sequences for the early and late genes. The early region encodes the regulatory proteins large tumor-antigen (LT-ag) and small tumor-antigen (st-ag), as well as other truncated LT-ag variants. The late region encodes the structural capsid proteins VP1, VP2 and VP3, and a regulatory protein named agnoprotein (reviewed in [16]). In addition, SV40 encodes a very late protein named VP4, which is involved in cell lysis [17]. VP4 corresponding open reading frames are present in the genomes of BKV and JCV, but the expression of this protein remains to be demonstrated.

The agnoprotein is a basic protein of 8 kD and its primary sequence is conserved among SV40, JCV and BKV especially at the N-terminal part of the protein [2]. The protein is phosphorylated *in vivo* [18]. Polyomavirus propagation studies with mutants in which phosphorylation sites were replaced by non-phosphorylatable alanines have been performed with BK and JC virus. JC virus expressing agnoprotein T21A, S7A/S11A/T21A or S7A/S11A is unable to propagate in SVG-A cells [19]. BK virus expressing agnoprotein S11A propagates in Vero and HUVECC cells, although less efficiently than wild-type [20]. Studies with wild-type and agnoprotein-deficient mutants of SV40, JCV and BKV revealed that the viruses which do not express agnoprotein propagate less efficiently but remain infectious [19]–[22].

Agnoprotein mainly resides in the cytoplasm, but a minor fraction can also locate to the nucleus in SV40, JCV and BKV infected cells [18], [20], suggesting functions in both cellular compartments. Previous studies showed the involvement of agnoprotein in nuclear egress, regulation of gene expression or/and replication, virion assembly or/and maturation and viral release [18], [22]–[24]. Recently, BKV agnoprotein was found to co-localize with lipid droplets, but the biological relevance of this finding is unclear [25]. Several cellular interaction partners have been identified for JCV agnoprotein, as reviewed in [2] and proteins of 50, 75 and 100 kD have been shown to precipitate with BKV agnoprotein [26]. However, the identity of these proteins and the biological functions of most of these interactions remain elusive.

α-soluble *N*-ethylmaleimide-sensitive fusion (NSF) attachment protein (α-SNAP) is a ubiquitous and indispensable component of the membrane fusion machinery. The plasma membrane and membrane-enclosed organelles like the endoplasmic reticulum (ER), Golgi apparatus and transport vesicles contain specialized membrane receptors known as soluble *N*-ethylmaleimide-sensitive factor attachment protein receptor (SNARE). During the fusion of two membranes, the SNAREs of opposing membranes interact in *trans* and undergo a conformational change by which they bring the two membranes in very close proximity. It is assumed that this proximity promotes fusion of the lipid bilayer and content mixing. After the fusion event, the SNARE complexes (now in *cis*) have to be disassembled in order to be ready to undergo another round of fusion. Therefore, α-SNAP binds *cis*-SNARE complexes, recruiting and stimulating the ATPase *N*-ethylmaleimide-sensitive factor (NSF) that disassembles *cis*-SNARE complexes [27], [28].

In an effort to improve our understanding of the role of agnoprotein in BKV's life cycle, we performed a yeast two-hybrid assay with BKV agnoprotein as bait and isolated putative cellular interaction partners, one of which was α-SNAP. We confirmed the interaction between BKV agnoprotein and α-SNAP both *in vitro* and *in vivo*. Moreover, we show that the N-terminal 38 amino acids of BKV agnoprotein are sufficient for this interaction. Finally, we provide evidence that the presence of ectopically expressed agnoprotein negatively interferes with secretion of VSVG-EGFP-reporter. We propose that agnoprotein modulates exocytosis.

## Materials and Methods

### Materials

Thrombin was obtained from Amersham Biosciences. Fetal Bovine Serum (FBS) and cell culture medium were obtained from Invitrogen (Carlsbad, CA, USA). CDP-star and MagicMark™ XP Western Protein Standard (CA, USA) and Invitrogen (Oslo, Norway), respectively. Antibodies against agnoprotein were described previously [26]. Antibodies against α-SNAP (FL-295), Hsp27 (H-77, sc-9012) and GST (Z-5; sc-459) were from Santa Cruz, while the antibody for actin was from Sigma Aldrich. SV40 LT-ag AB-2 was from Calbiochem, while antiserum for BKV LT-ag and VP1 has been described previously [29],[30]. Alkaline phosphatase conjugated anti-rabbit antibody was purchased from DAKO (Denmark). Fluorophore-conjugated antibodies Alexa Fluor 488 and 568 were obtained from Invitrogen Life Technologies (Oslo, Norway).

The agnoprotein peptides 1–37 (MVLRQL SRQASVKVGKTWTGTKKRAQRIFIFILELLL), 15–45 (GKTWTGTKKRAQRIFIFILE LLLEFCRGED) and 38–66 (EFCRGEDSVDGKNKSTTALPAVKDSVKDS) were synthesized by Gene Cust (Luxembourg).

### Cell lines

HEK293 (ECACC catalog number 85120602) was purchased from European Collection of Cell Cultures, while Vero (ATCC catalog number CCL81) and U2OS cells (ATCC catalog number HTB-96) were purchased from American Type Culture Collection. All the cells were maintained in Dulbecco's modified Eagle's medium supplemented with 10% (v/v) fetal bovine serum, penicillin (100 units/ml), and 100 µg/ml streptomycin (Invitrogen) in a CO_2_ incubator (5% CO_2_) at 37°C.

### Plasmid constructs

The empty expression vectors pRcCMV, pCMV and pGEX-4T-1 were purchased from Invitrogen, Clontech and GE Healthcare, respectively. pRcCMV-agno and pGST-agno have been described previously [20], [26]. pCMV5-α-SNAP was purchased from Origen (Rockville, USA), while pET11d-His-α-SNAP was provided by Dr. Mitsuo Tagoya [31], and the VSVG-EGFP ts construct [32] was purchased from Addgene (Cambridge, USA). pCMV2-Flag-Hsp27 was kindly provided by Dr. Kuy-Jin Park [33]. pGST-agno-stop38 was created by introducing a stop codon by site-specific mutagenesis using a complementary primer set of 5′-gag ctt ttg ctg taa ttt tgt aga ggt g-3′ (only one strand shown). The mutation was confirmed by sequencing, and the obtained construct was used as template in a new round of site-specific mutagenesis with the complementary primer set of 5′-ctt ttg ctg taa tag tga tga ggt gaa gac-3′ (only one strand shown) resulting in additional three successive stop codons. Finally, all mutations were verified by sequencing.

### Protein purifications

GST fusion proteins were purified from *Escherichia coli* BL21 extracts using glutathione-agarose beads, and the agnoprotein moiety was enzymatically cleaved from the GST-beads with thrombin according to the instructions of the manufacturer. His-tagged proteins were purified from *Escherichia coli* BL21 or BL21(DE3) according to the instructions of the manufacturer.

### Preparation of viral genome and transient transfection

Transfection studies with expression plasmids in HEK293 and Vero cells were done using Lipofectamine 2000 (Invitrogen) according to the instructions of the manufacturer. BKV DNA used in transfection was prepared from the plasmid pBK34-2 (ATCC cat. no. 45025) as follows. The plasmid was digested with BamHI/BglII to release the BKV genome which is cloned in the BamHI site of pB322, while BglII digested pBr322 in several smaller fragments. The linearized DNA was purified and relegated with T4-ligase and used for transfection of U2OS cells.

### Immunoprecipitations

Confluent 10 cm dishes with HEK293 cells were transfected with appropriate expression plasmids. The cells were harvested and the proteins immunoprecipitated as previously described [34]. For the *in vitro* co-immunoprecipitations, purified protein(s) were added to 200 µl precipitation buffer containing 50 mM Tris-HCl, pH 8.0, 150 mM NaCl, 1 mM EDTA, 1 mM DTT and complete protease inhibitor mixture (Roche Diagnostic) and incubated at room temperature for 10 minutes. The samples were precleared by addition of 50 µl 50% protein G-Sepharose slurry followed by 30 minutes incubation at room temperature. The supernatant was then incubated with the appropriate antibody for 1 hour at 4°C, followed by precipitation of the complexes with protein G-sepharose. The immune complexes were washed three times with an extensive volume of ice cold precipitation buffer (see above), and subsequently subjected to SDS PAGE followed by etiher immunoblot analysis Coomassie blue G staining.

### GST-pulldown

3×10^6^ HEK293 cells were washed twice with PBS, and harvested in 3 ml ice-cold PBS with 1% Triton-X100 (PBST). The GST-pulldown was performed as described previously [35]. Briefly, the cell lysate was precleared with 25 µg GST and 50 µl of 50% slurry of glutathione-agarose beads for 2 hours at 4°C. An input control was taken, and the cell lysate was divided into three tubes and 10 µg of GST, GST-agnoprotein or GST-agnoprotein-stop38 were added. The GST proteins were recovered after addition of glutathione beads and washed extensively in PBST. The proteins were eluted from the beads by addition of 20 µl loading buffer (1×LDS-buffer with 100 mM DTT) followed by 10 minutes incubation at 70°C. The GST proteins were subsequently subjected to immunoblot analysis.

### Immunofluorescence analysis

Cells were rinsed twice with phosphate-buffered saline (PBS) and fixed for 10 minutes with 4% formaldehyde. Next, the cells were washed twice with PBS and permeabilized with PBS containing 0.1% Triton X-100 for 5 minutes. The cells were thereafter washed twice with cold PBS prior to blocking for 30 minutes with 3% goat serum in PBS at room temperature. Finally immunostaining was performed, i.e. PBS with 1% goat serum and primary antibodies (rabbit antiserum against agnoprotein (1∶600 dilution) and/or mouse antibody against α- SNAP (1∶100) were added to the cells, and incubated at 37°C for 30 minutes. After seven washes with PBS, the cells were stained with secondary rabbit antibody conjugated to Alexa Fluor 488 (green) (1∶500) or secondary mouse monoclonal antibody conjugated to Alexa Fluor 568 (red) (1∶500) in 1% goat serum in PBS for 30 minutes at room temperature. After seven new washes with PBS, the cells were stained with DRAQ5 (1∶1000) for 5 minutes at room temperature. The cells were finally washed twice in PBS, and examined using a confocal laser-scanning Zeiss LSM 510 META and a Leica SP5 microscope.

### Immunoblot analysis

Immunoblot analyses were performed on cell extracts as described previously [20].

### Yeast two-hybrid screen

A yeast two-hybrid service was ordered from www.panbionet.com. Briefly, the BKV agnoprotein gene was cloned in frame with the coding sequence of the DNA binding domain of GAL4(GAL4DB) in pGBKT vector. GAL4-BKV-agnoprotein was then used as bait in a human kidney cDNA library and a human thymus cDNA library in the yeast strain PBN204, which has *ADE2*, *URA3* and *lacZ* genes as reporters. The transformants were spread on selection media (SD-LWU) where the selection was based on the interaction between bait and prey. Thereafter, the yeast colonies on uracil deficient media were analyzed for other reporter expression (lacZ and ADE2) that is under the control of a GAL4 promoter. This approach yielded a total of 6 prey candidates, which showed agnoprotein-dependent reporter gene expression.

## Results

### BKV agnoprotein and α-SNAP partly co-localize in mammalian cells

The function(s) of BKV agnoprotein remains incompletely understood. In order to explore the role of agnoprotein, a yeast two-hybrid screen with BKV agnoprotein as bait against human kidney and thymus cDNA libraries was performed. The two screens yielded a total of 6 putative interaction partners for BKV agnoprotein. One of the interaction partners was found in both libraries, while the others were found in only one of the libraries. Among the interaction partners identified in the human kidney library was α-soluble *N*-ethylmaleimide-sensitive fusion (NSF) attachment protein (α-SNAP).

**Figure 1:**
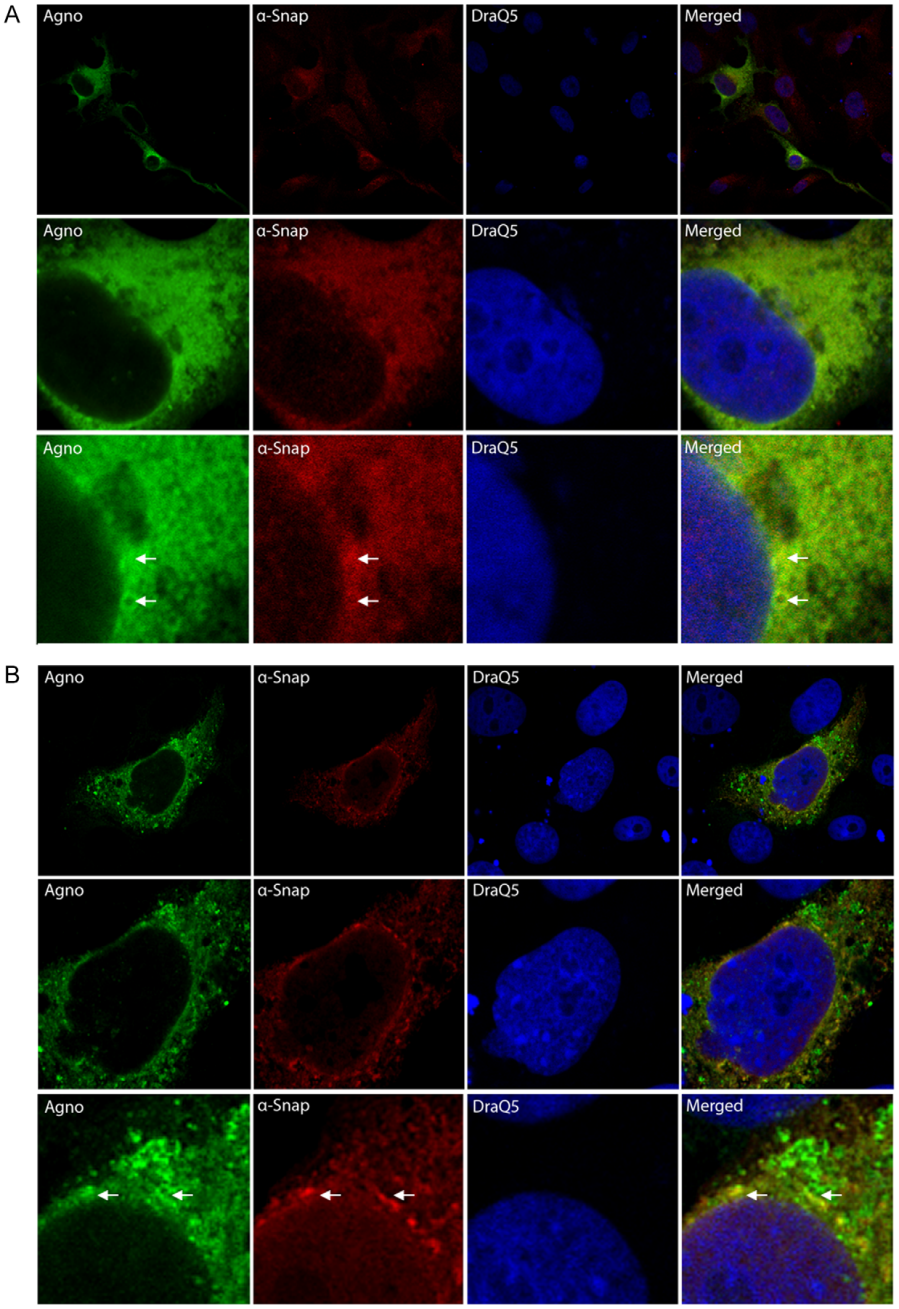
BKV agnoprotein and α-SNAP partly co-localize in Vero cells. (A) Vero cells were transfected with an expression plasmid encoding BKV agnoprotein (200 ng). The cells were fixed with paraformaldehyde and immunostained with polyclonal rabbit antibodies against BKV agnoprotein and monoclonal mouse antibodies against endogenous α-SNAP. The primary antibodies were detected by addition of mouse Alexa-568-coupled secondary antibody (red) and rabbit Alexa488-coupled antibody (green), and the cells were evaluated by confocal microscopy. The cell nuclei were visualized by DRAQ5 staining. The middle and lower panel is an enlargement of a cell shown in upper panel. (B) Vero cells were co-transfected with expression plasmids encoding BKV agnoprotein (200 ng) and α-SNAP (200 ng). The cells were fixed and stained as described in A. The middle and lower panel is an enlargement of the cell shown in upper panel.

If the proteins would be able to interact *in vivo*, they should at least have partly overlapping localization within mammalian cells. Therefore, we first addressed the localization of BKV agnoprotein and α-SNAP in mammalian cells. Thereto, Vero cells were transfected with an expression plasmid encoding BKV agnoprotein and immunostained with antibodies against BKV agnoprotein and α-SNAP. The nucleus was visualized by DRAQ5-staining. BKV agnoprotein and the endogenous α-SNAP localize diffusely throughout the cytoplasm. Some common areas are neither stained by antibodies against agnoprotein nor α-SNAP. Despite the rather diffuse staining, a partial overlap of the signals of agnoprotein and α-SNAP was observed (Figure 1A and results not shown). In order to corroborate this result, we ectopically expressed both BKV agnoprotein and α-SNAP in Vero cells. When both proteins were over-expressed within the cells, BKV agnoprotein localized predominantly perinuclear and throughout the cytoplasm (Figure 1B, figure S1 and results not shown). Staining against α-SNAP revealed again a partly similar pattern as cells stained against BKV agnoprotein, suggesting partial co-localization (Figure 1B, and results not shown). These results demonstrate that the two proteins can localize to the same area within the eukaryotic cell suggesting that a physical interaction may occur *in vivo*.

### BKV agnoprotein interacts specifically and directly with α-SNAP

**Figure 2:**
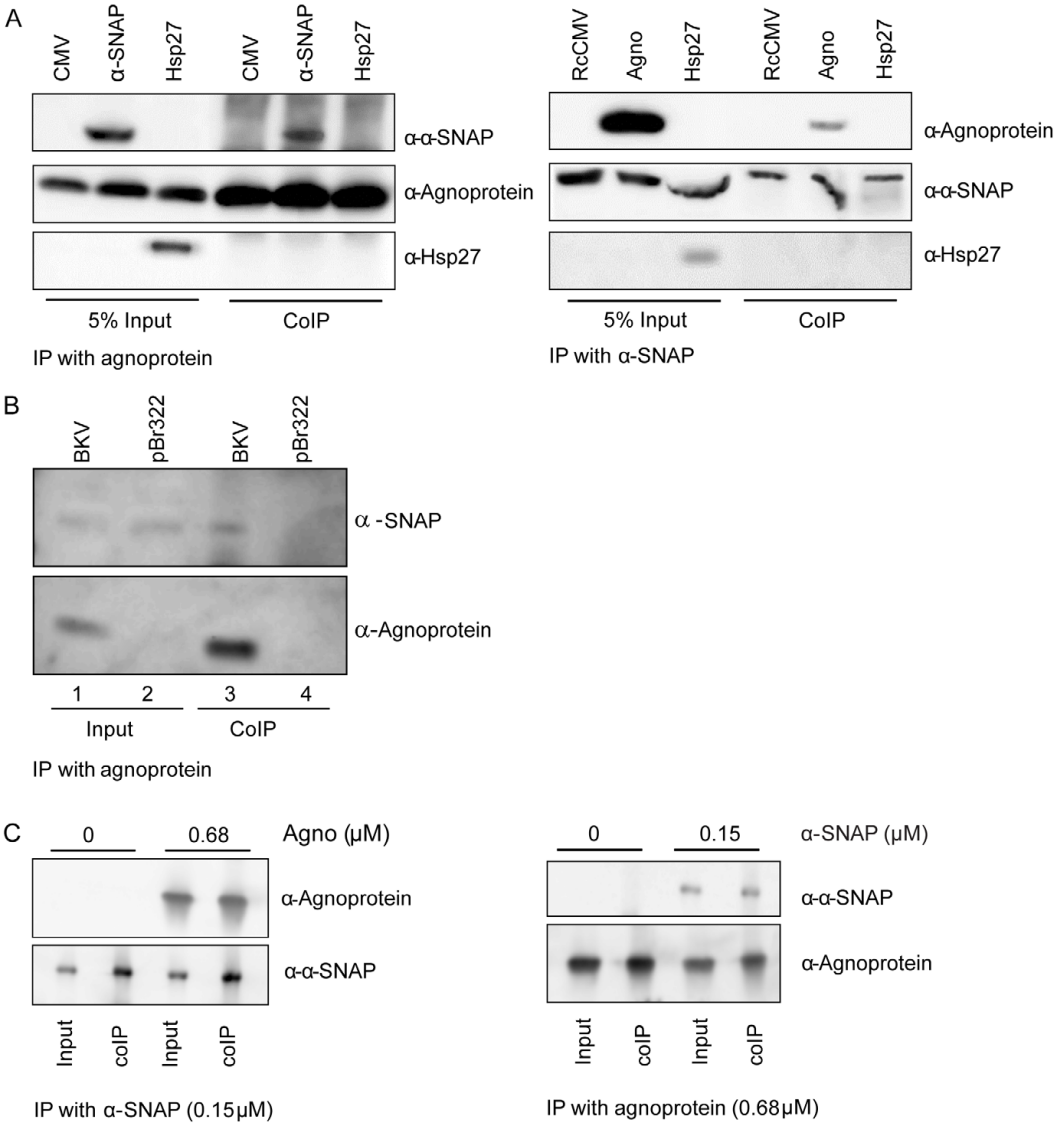
BKV agnoprotein specifically interacts with α-SNAP. (A) HEK cells were transfected with 8 µg of expression plasmids encoding BKV agnoprotein, α-SNAP and Hsp27, or 8 µg of the empty vector pCMV5 (left panel), or 8 µg of expression plasmids encoding α-SNAP, BKV agnoprotein and Hsp27 or 8 µg of the empty vector pRcCMV (right panel). Whole cell extract was immunoprecipitated with anti-agnoprotein and anti-α-SNAP antibodies, respectively. The precipitated proteins were separated by SDS PAGE followed by western blot. The immunoprecipitated proteins were detected using anti-agnoprotein (left panel, middle section) and anti- α-SNAP antibodies (right panel, middle section), respectively. The co-immunoprecipitated proteins were detected with antibodies against α-SNAP and Hsp27 (left panel, upper and lower section) or agnoprotein and Hsp27 (right panel, upper and lower section), respectively. (B) U2OS cells were seeded into two 10 cm dishes and transfected with 8 µg purified BKV genome or pBr322. Cell lysates were prepared 48 hours post transfection and agnoprotein was immunoprecipitated. The immunoprecipitated complexes were separated on SDS PAGE and immunoblotted with antibodies against BKV agnoprotein (lower panel) and antibodies against α-SNAP (upper panel). (C) Purified His-α-SNAP (0.15 µM) or BKV agnoprotein (0.68 µM) were left untreated or supplied with purified 0.68 µM BKV agnoprotein or 0.15 µM α-SNAP, respectively, and immunoprecipitated with antibodies against α-SNAP or BKV agnoprotein, respectively (lower panel, both sections). The immunocomplexes were separated by SDS PAGE, and the co-immunoprecipitated proteins were assayed by immunoblotting with antibodies against agnoprotein or α-SNAP, respectively (top panel, both sections).

In order to evaluate whether BKV agnoprotein interacts with α-SNAP in mammalian cells, HEK293 cells were transfected with an expression plasmids encoding BKV agnoprotein and/or α-SNAP or the empty vector. Using antibodies against BKV agnoprotein and α-SNAP, we performed reciprocal co-immunoprecipitations and detected subsequent precipitates by immunoblot. Ectopically expressed α-SNAP was successfully precipitated by BKV agnoprotein (Figure 2A, left panel, upper section, lane 5), while α-SNAP antibodies could co-immunoprecipitate BKV agnoprotein (Figure 2A, right panel, upper section, lane 5). As an additional control for the specificity of the BKV agnoprotein- α-SNAP interaction, we chose to include a control where we over-expressed Hsp27 and either BKV agnoprotein or α-SNAP prior to reciprocal co-immunoprecipitation. As seen in the lower section, both panels in Figure 2A, ectopically expressed Hsp27 is detected in the input controls, but is not co-immunoprecipitated with neither BKV agnoprotein nor α-SNAP (Figure 2A, lower section, both panels).

Next, we asked whether this interaction would also occur under physiological conditions during viral BKV propagation. Because BKV can persistently infect U2OS cells [36] and the transfection efficiency of these cells is 30–40%, U2OS cells were chosen for the experiment. Cells were transfected with either the complete BKV genome or the empty vector pBr322. The cells that are transfected with BKV genome will express viral proteins. Time-course experiments had shown that BKV agnoprotein expression is clearly detectable 48 hours after transfection (results not shown), and the cells were therefore harvested at this time point. Immunoprecipitations were performed using anti-agnoprotein antibodies, and the precipitated proteins were separated by SDS PAGE followed by immunoblotting with antibodies against α-SNAP. We detected endogenous α-SNAP in the cell lysates from both BKV- or pBR322-transfected U2OS cells (Figure 2B, lanes 1 and 2). Similarly, BKV- but not pBR322-transfected U2OS cells expressed agnoprotein (Figure 2B, lanes 1 and 2). The antibody against BKV agnoprotein precipitated agnoprotein and α-SNAP in BKV-transfected but not in pBr322-transfected U2OS cells (Figure 2B, lanes 3 and 4). Considering that approximately 30–40% of the cells become transfected with BKV genome and expresses BKV agnoprotein, and the low level of endogenous α-SNAP in these cells, complexes of both proteins could still be detected by co-immunoprecipitation. This suggests that α-SNAP and BKV agnoprotein can interact at physiological concentrations during viral infection.

One limitation with co-immunoprecipitations - or yeast two-hybrid experiments - is that the interaction might not be direct, but is mediated via other protein(s) that are in the precipitated complex. To verify a direct interaction between BKV agnoprotein and α-SNAP, we purified BKV agnoprotein and His-tagged α-SNAP protein, and incubated the proteins together. Then reciprocal *in vitro* co-immunoprecipitation experiments were performed. The immunoprecipitated α-SNAP could clearly co-precipitate BKV agnoprotein (Figure 2C, left panel). Similarly, BKV agnoprotein could co-immunoprecipitate α-SNAP (Figure 2C, right panel).

In conclusion, BKV agnoprotein can interact directly with α-SNAP both *in vitro* and *in vivo* with ectopically or endogenously expressed proteins.

### The N-terminal part of BKV agnoprotein can interact with α-SNAP

**Figure 3:**
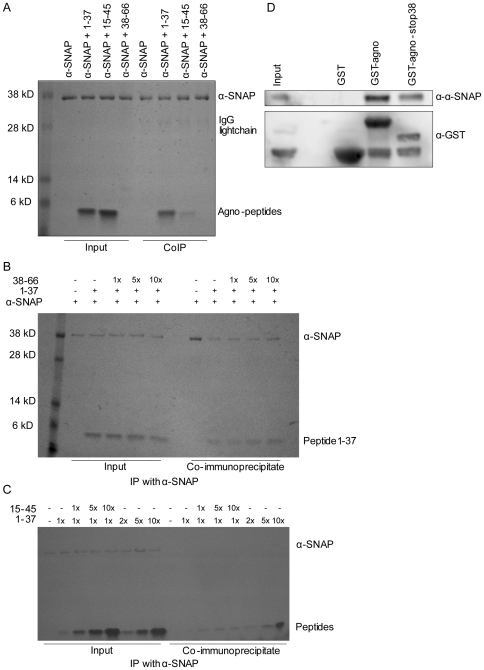
The N-terminal 38 amino acids of BKV agnoprotein are predominantly required for the interaction with α-SNAP. (A) Purified His-α-SNAP (3 µM) was left untreated or supplied with peptide 1–37 (25 µM), peptide 15–45 (25 µM) or peptide 38–66 (25 µM). Antibodies against α-SNAP were added, and the immunocomplexes were separated by SDS PAGE and stained with Coomassie blue. (B) Purified His-α-SNAP (750 nM) was left untreated or supplied with 6.25 µM peptide 1–37 and ratios 1∶0, 1∶1, 1∶5 and 1∶10 of peptide 38–66. α-SNAP was immunoprecipitated by use of anti-α-SNAP antibodies, and the obtained immunocomplexes were separated by SDS PAGE and stained with Coomassie blue. (C) Purified His-α-SNAP (750 nM) was left untreated or supplied with 6.25 µM peptide 1–37 and ratios 1∶0, 1∶1, 1∶5 and 1∶10 of the peptides 15–45 or peptide 1–37. α-SNAP was immunoprecipitated by use of anti-α-SNAP antibodies, and the obtained immunocomplexes were separated by SDS PAGE and stained with Coomassie blue. (D) Proteins (10 µg GST, GST-agnoprotein or GST-agnoprotein-stop38) expressed and purified from bacteria were used to pull down endogenous α-SNAP from HEK293 cells. The level of α-SNAP which was pulled down was evaluated by immunoblot using antibodies against α-SNAP (upper panel). In order to evaluate the level of GST proteins, the membrane was stripped and reprobed with antibodies against GST (lower panel).

In order to evaluate which region of agnoprotein is involved in the interaction with α-SNAP, peptides consisting of various parts of BKV agnoprotein were synthesized. Peptide 1 consists of amino acids 1–37, while peptide 2 and 3 encompass amino acids 15–45 and 38–66, respectively. Our polyclonal agnoprotein antibody seems to detect the various peptides with different efficiency (results not shown). For the *in vitro* binding experiments, we therefore incubated 25 µM peptides with 3 µM α-SNAP, and precipitated protein-peptide complexes using antibodies against α-SNAP. The precipitated complexes were separated on SDS PAGE followed by Coomassie blue staining (Figure 3A). Agnopeptide 1–37 and agnopeptide 15–45 are seen as clear bands of approximately equal strength in the input control. Unfortunately, peptide 38–66 was not detected on Coomassie blue, Ponceau S or silver-stained gels (Figure 3A and results not shown), however the peptide's integrity is confirmed by immunoblot and HPLC (Figure S2 and results not shown). Both peptides 1–37 and 15–45 co-immunoprecipitated with α-SNAP, although the former did so more potently (Figure 3A). In order to evaluate whether the peptide 38–66 also could interact with α-SNAP, we decided to perform a competitive *in vitro* co-immunoprecipitation. To this end, α-SNAP and peptide 1–37 were mixed in the presence of increasing concentrations of peptide 38–66. If the latter peptide interacts with α-SNAP, then the interaction between α-SNAP and peptide 1–37 should be reduced in the presence of increasing levels of peptide 38–66. As seen in Figure 3B, increasing levels of peptide 38–66 do not influence the ability of peptide 1–37 to co-immunoprecipitate with α-SNAP, suggesting that the C-terminal part of agnoprotein is not involved in the interaction with α-SNAP. Next, we decided to perform a similar *in vitro* co-immunoprecipitation using peptide 1–37 and increasing concentrations of 15–45 or 1–37 as a control. Peptide 1–37 and increasing amount of peptide 15–45 or 1–37 are clearly seen in the input controls (Figure 3C, lane 2–8). The antibodies against α-SNAP could efficiently co-immunoprecipitate peptides from the mixture containing peptide 1–37 and increasing level of peptide 15–45, however, the level of co-immunoprecipitated peptides do not correlate with the increase in peptide 15–45 concentration (Figure 3C, lane 11–13). In contrast, the level co-immunoprecipitated peptide 1–37 correlated well with the increasing amount of peptide 1–37 (Figure 3C, lane 14–16). The lack of correlation of co-immunopreciptitated 15–45 peptide with increasing concentration of the peptide, may suggest that 1–37 peptide binds α-SNAP. This is in agreement with the experiment where α-SNAP pulled down more 1–37 peptide compared to 15–45 peptide (Figure 3A). This finding suggests that the 1–15 area of BKV agnoprotein is of particular importance in the interaction to α-SNAP.

As another approach to evaluate the involvement of the N-terminal part of BKV agnoprotein in the interaction with α-SNAP, we decided to generate a C-terminal truncated variant of agnoprotein by introducing four stop codons starting at amino acid 38 of BKV agnoprotein. Purified GST, GST-agnoprotein and GST-agnoprotein-stop38 were then evaluated for their ability to pull down endogenous α-SNAP from HEK293. The loading of GST fusion proteins was evaluated by GST-antibodies (Figure 3D, lower panel). GST is seen in all the GST-pulldown experiments, and also slightly in the input control as expected (see Materials and Methods). The level of GST-agnoprotein-stop38 is slightly lower than GST-agnoprotein. Still, both GST-agnoprotein and GST-agnoprotein-stop38 pulled down endogenous α-SNAP from the cell lysate. No α-SNAP was detected when GST was used as bait, confirming the involvement of BKV agnoprotein in the pulldown (Figure 3D, upper panel).

In summary, these results strongly suggest that the conserved N-terminus of BKV agnoprotein is predominantly involved in the binding of α-SNAP.

### BKV agnoprotein negatively influences the secretion of VSVG-EGFP

α-SNAP is involved in disassembly of *cis*-SNARE complexes after membrane fusion along the secretory pathway [28]. A marker that is commonly used to study the constitutive secretory pathway is the Vesicular stomatitis virus glycoprotein ts045 tagged with green fluorescent protein (VSVG-EGFP) [32]. At restrictive temperature, i.e. 40°C, VSVG-EGFP adopts a reversible abnormally folded structure, resulting in its retention in the ER. Transfer to the permissive temperature of 32°C, allows the protein to fold correctly and to exit the ER and travel through the Golgi apparatus to the plasma membrane [32].

**Figure 4:**
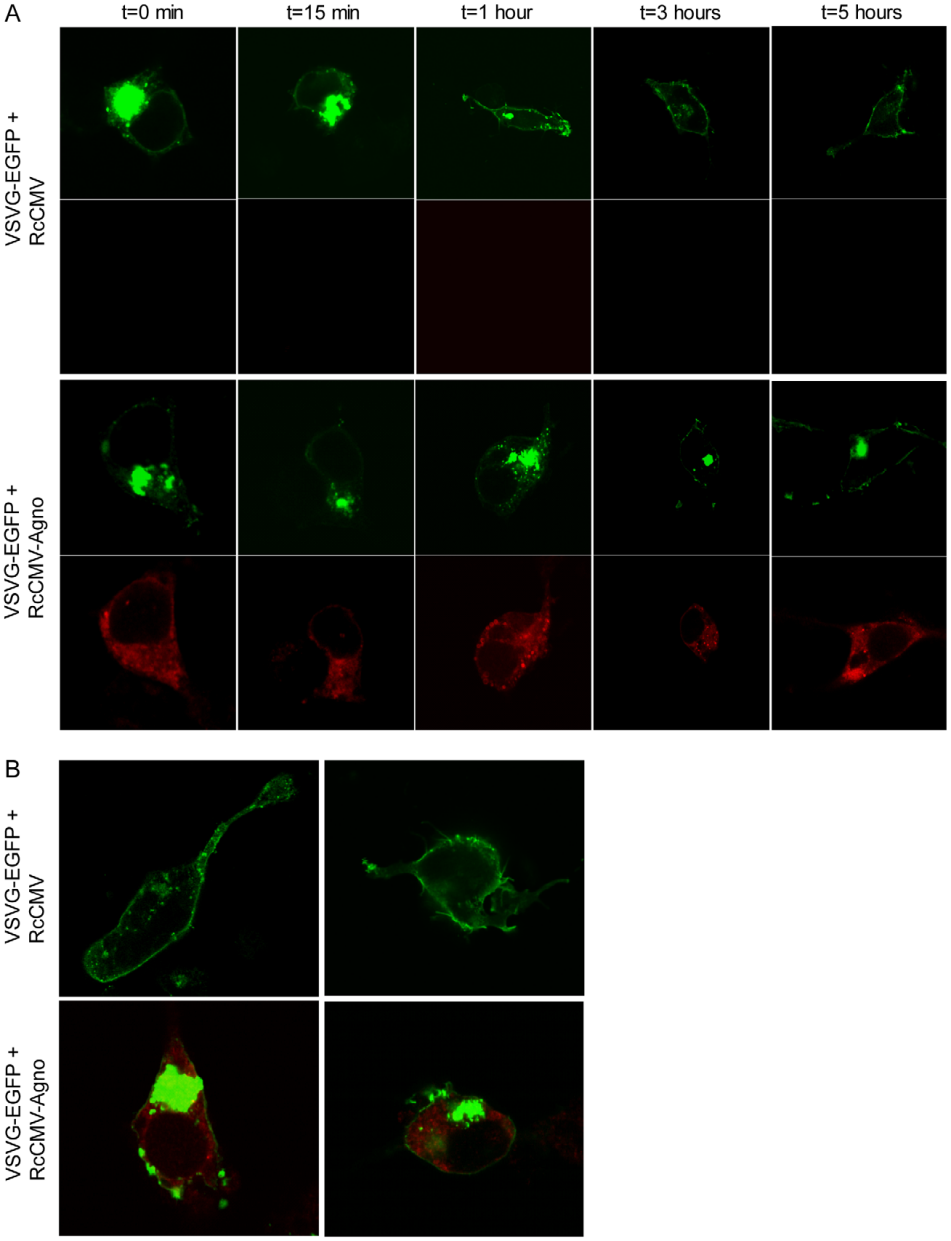
BKV agnoprotein negatively influences the transport of VSVG-EGFP. (A) HEK293 cells were transfected with VSVG-EGFP(ts045) and RcCMV or RcCMV-agnoprotein and incubated at 40°C overnight. The cells were transferred to 32°C, fixed and immunostained against BKV agnoprotein after various time intervals (0, 15 minutes, 1 h, 3 h and 5 h). The subcellular localization of VSVG-EGFP (green) in absence or presence of BKV agnoprotein (red) was evaluated by confocal microscopy. (B) Enlargement of HEK293 cells transfected with VSVG-EGFP(ts045) and RcCMV or RcCMV-agnoprotein and treated as in A. The cells were fixed and immunostained after 5 hours, and evaluated by confocal microscopy.

As we typically obtained transfection efficiency of 80–90% in HEK293 cells, we chose this cell line to investigate the transport of VSVG-EGFP in the absence or presence of BKV agnoprotein. HEK293 cells were transfected with expression plasmids encoding VSVG-EGFP and BKV agnoprotein or pRcCMV and incubated at 40°C over night. The next day, cells were fixed immediately (t = 0) and immunostained for BKV agnoprotein, while the rest of the cells were transferred to 32°C, captured by fixation and immunostained at various time intervals (t = 15 min, t = 1 h, t = 3 h and t = 5 h). For each condition, the localization of VSVG-EGFP in the cells was evaluated by confocal microscopy for which at least 20 cells were scored per condition to clearly establish the respective VSVG-EGFP localization. At t = 0, VSVG-EGPF was trapped within the cell as expected in all cells (Figure 4A). Similar patterns were also found at t = 15 minutes and after 1 hour in cells in presence or absence of BKV agnoprotein (Figure 4A). After 3 and 5 hours, the cells without BKV agnoprotein expression lack the concentrated VSVG-EGFP within the cell, and more fluorescence appeared at the plasma membrane (Figure 4A and B). In contrast, the VSVG-EGFP remained trapped within the cells expressing BKV agnoprotein (Figure 4A and B). These results suggests that BKV agnoprotein negatively influences the export of VSVG-EGFP to the plasma membrane.

## Discussion

In this study, we have identified the first cellular interaction partner for BKV agnoprotein. The interaction between this viral protein and α-SNAP has been shown by several methods including GST-pull downs, co-immunoprecipitation *in vitro* and from cell lysates with ectopic or endogenous levels of the proteins. We have also shown that the proteins can co-localize within the cells. Moreover, we found that the amino acids 1–37 of agnoprotein are predominantly involved in the interaction with α-SNAP. Finally, we demonstrated that expression of BKV agnoprotein negatively influences the secretory pathway by blocking protein export within the cell, as evaluated by the VSVG-EGFP reporter.

So far, few interaction partners have been identified for JCV agnoprotein and none for BKV agnoprotein. Although, BKV agnoprotein pulled down protein(s) with molecular masses of approximately 50, 75 and 100 kD from BKV-infected HUV-EC-C cells [26], the identity of these proteins remains unknown. Yeast two-hybrid assays have yielded two interaction partners for JCV agnoprotein. The highly conserved N-terminal 24 amino acids of JCV agnoprotein was used as bait in human embryonic kidney 293 cells and yielded HP1-α which was found to be involved in nuclear egress of viral particles [24]. Our yeast two-hybrid screen did not yield HP1-α as an interaction partner to BKV agnoprotein, however this may be due to various sources and quality of the cDNA libraries. Another yeast two-hybrid screen using full-length JCV agnoprotein as bait in a human brain cDNA library resulted in the discovery of FEZ-1 as a brain-specific partner for JCV agnoprotein [37]. Our yeast two-hybrid screen using two different human libraries identified 6 new putative partners not previously identified as partners for BKV, JCV or SV40 agnoprotein. The cellular interaction partner(s) of agnoprotein may therefore depend on the type of virus, cell line and perhaps also the concentration of agnoprotein as well as post-translational modifications of agnoprotein and/or its putative partner.

BKV agnoprotein has previously been found to co-localize with lipid droplets [25]. The proteome of lipid droplets includes the SNAREs SNAP23, syntaxin-5 and VAMP4 as well as NSF and α-SNAP [39], [40]. Here we describe that BKV agnoprotein interacts with α-SNAP, which therefore could be the direct interaction partner localized on the lipid droplets. Interestingly, fusion of lipid droplets required α-SNAP and SNAREs [38], and these proteins, present on vesicles and target compartments, ensure appropriate fusion and subsequent disassembly [27], [28]. As overexpression of BKV agnoprotein negatively influences the transport of VSVG-EGFP (Figure 4), it is possible that the presence of BKV agnoprotein may impair the disassembly process through competition with NSF for binding to α-SNAP. Such a reduction in disassembly would limit the availability of SNAREs, thereby causing the inhibition of secretion or fusion of lipid droplets.

Agnoprotein is highly conserved among SV40, JCV and BKV, especially within the 35–40 amino acids at the N-terminal part [18]. Interestingly, agnopeptide 1–37 and GST-agnoprotein-stop38 could both specifically interact with α-SNAP. Given the sequence conservation in the N-terminal region of agnoprotein, our data suggest that agnoprotein of JCV and SV40 may also interact with α-SNAP and that the inhibitory mechanism may be conserved as well.

BKV agnoprotein has been found to be phosphorylated *in vivo* [20], [26], and Ser-7, Ser-11 and Thr-21 were identified as phospho-acceptor sites for PKC and PKD *in vitro* [20]. Circular dichroism results of BKV agnoprotein and variants where Ser-11 was mutated into alanine or aspartate suggest no major differences in secondary structure (results not shown). Moreover, BKV agnoprotein mutants where Ser-11 or Ser-7/Ser-11/Thr-21 was replaced by non-phosphorylatable alanine could still interact with α-SNAP, as evaluated by co-immunoprecipitation of cell lysates ectopically expressing these proteins (results not shown). These results could indicate that the interaction between α-SNAP and BKV agnoprotein is not regulated by phosphorylation at least not by these phospho-acceptor sites combinations. α-SNAP may also be regulated by phosphorylation [40], [41], however, we have not tested whether phosphorylation of α-SNAP is required for its interaction with agnoprotein.

Several hypotheses could be made for the functional consequence(s) of the interaction between agnoprotein and α-SNAP. Firstly, agnoprotein may inhibit secretion and thereby interfere with cell membrane integrity. JCV agnoprotein has been found to follow an exocytic route from ER to plasma membrane, where it can function as viroporin [22]. By its interaction with α-SNAP and its ability to interfere with secretion, agnoprotein may regulate the formation of viroporins. Secondly, agnoprotein may negatively influence secretion as a strategy for immune evasion. Cells continuously degrade and present peptides via major histocompatibility complex I (MHC) molecules. The assembly of MHC I and the antigenic peptide occurs in the ER, and the complexes are transported to the cell surface by the constitutive secretory pathway. At the cell surface, MHC I presents antigenic peptides to cytotoxic T cells which results in destruction of infected cells [42], [43]. Several viruses interfere with the antigen presentation pathway. Some viruses interfere with proteasomal processing of antigenic peptides within the cytosol, while other viruses interfere with peptide transport across the lumen of the ER or with the retention, dislocation and destruction of MHC I in the ER and early secretory compartments [44]. Another strategy used by certain Herpesvirus subfamilies is to increase endocytosis and subsequent degradation of MHC class I molecules from the membrane ([45] and references therein), while poliovirus chooses to inhibit the secretory pathway by means of viral proteins 2B or 3A, where the latter exhibits a stronger effect and is specific for ER-to-Golgi traffic [46]–[48]. Alternatively, an agnoprotein-mediated inhibition of the secretory pathway may reduce the secretion of cytokines and/or α- and β-interferons. This strategy is used by poliovirus where protein 3A limits IL-6, IL-8 and β-interferon secretion during viral infection [47]. Another purpose of agnoprotein to perturb the secretory pathway through targeting α-SNAP could be to prevent premature release of incomplete virions. In the initial stage of the late phase, agnoprotein is abundantly expressed, and this may inhibit secretion of capsid proteins and viral DNA that are incompletely assembled into infectious virions. Later, when agnoprotein expression decreases, the inhibition of secretion is abrogated and completely maturated virus particles can be released.

Agnoprotein is a small auxiliary protein, which has been found to influence viral transcription, as well as viral assembly, viral release and propagation [18]. The viral protein is expressed late in the viral life cycle, and the function may depend on the concentration and post-transcriptional regulation of itself, as well as its interaction partner. The interference with secretion may therefore vary during viral propagation, but this remains to be investigated.

In summary, we have identified the first cellular interaction partner of BKV agnoprotein, and we have shown that the interaction between α-SNAP and agnoprotein occurs in BKV infected cells. The N-terminal 1–37 amino acids are involved in the interaction, which may suggest that also JCV and SV40 agnoprotein can interact with α-SNAP. Finally, we show that BKV agnoprotein negatively interferes with secretion.

## Supporting Information

Figure S1 **Vero cells were transfected with expression plasmids encoding BKV agnoprotein (200 ng) and α-SNAP (200 ng).** The cells were fixated and immunostained against BKV agnoprotein and α-SNAP. The cell nuclei were visualized by DRAQ5 staining. Arrows indicate co-localization of BKV agnoprotein and α-SNAP.
(TIF)

Figure S2 **Peptide 38–66 is detected by immunoblot.** 50 ng, 250 ng, and 500 ng peptide 38–66 were separated on SDS PAGE (NuPAGE, Invitrogen), followed by immunoblot using antibody against agnoprotein as primary antibody, and HRP-conjugated goat anti rabbit secondary antibody.
(TIF)
