# Supplementary figures and images for: BKV Agnoprotein Interacts with α-Soluble N-Ethylmaleimide-Sensitive Fusion Attachment Protein, and Negatively Influences Transport of VSVG-EGFP
Source: PLoS One. 2011 Sep 12;6(9):e24489. doi: 10.1371/journal.pone.0024489 (PMC3171462; doi:10.1371/journal.pone.0024489)

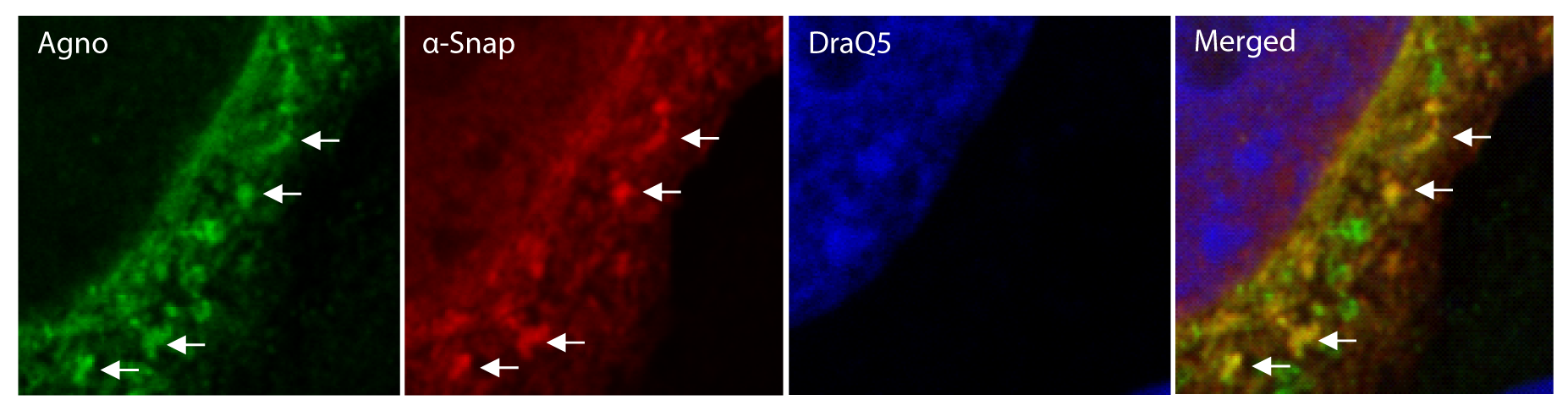

Supplement: Figure S1 — Vero cells were transfected with expression plasmids encoding BKV agnoprotein (200 ng) and α-SNAP (200 ng). The cells were fixated and immunostained against BKV agnoprotein and α-SNAP. The cell nuclei were visualized by DRAQ5 staining. Arrows indicate co-localization of BKV agnoprotein and α-SNAP. (TIF) [file pone.0024489.s001.tif]

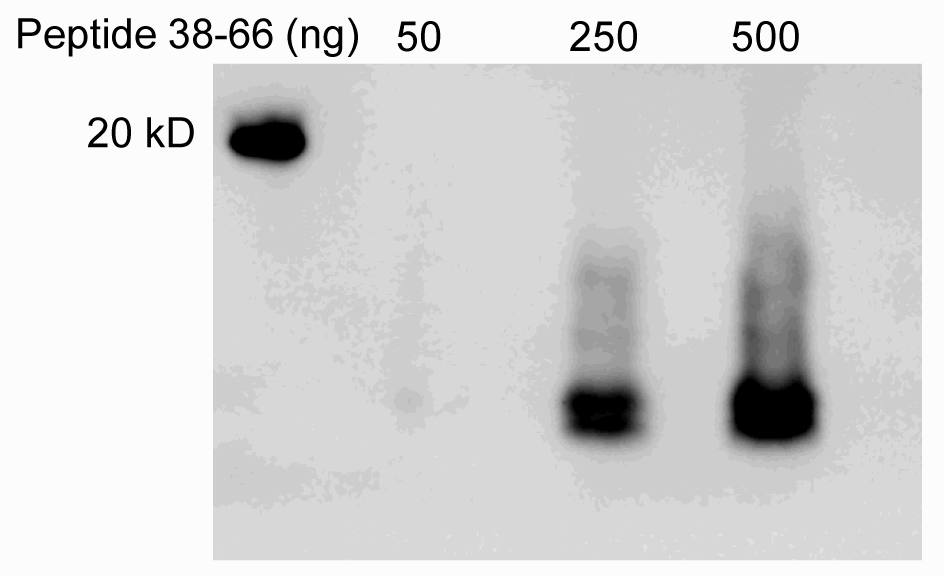

Supplement: Figure S2 — Peptide 38–66 is detected by immunoblot. 50 ng, 250 ng, and 500 ng peptide 38–66 were separated on SDS PAGE (NuPAGE, Invitrogen), followed by immunoblot using antibody against agnoprotein as primary antibody, and HRP-conjugated goat anti rabbit secondary antibody. (TIF) [file pone.0024489.s002.tif]
